# Observer training for computer-aided detection of pulmonary nodules in chest radiography

**DOI:** 10.1007/s00330-012-2412-7

**Published:** 2012-03-25

**Authors:** Diederick W. De Boo, François van Hoorn, Joost van Schuppen, Laura Schijf, Maeke J. Scheerder, Nicole J. Freling, Onno Mets, Michael Weber, Cornelia M. Schaefer-Prokop

**Affiliations:** 1 Department of Radiology, Academic Medical Centre (AMC), Meibergdreef 9, 1105 AZ Amsterdam, The Netherlands; 2 Department of Radiology, General Hospital Vienna, University Hospital, Vienna, Austria; 3 Department of Radiology, Meander Medical Center, Amersfoort, The Netherlands

**Keywords:** Radiographic image interpretation, Computer-assisted, Solitary pulmonary nodule, Radiography, Lung, Education

## Abstract

**Objectives:**

To assess whether short-term feedback helps readers to increase their performance using computer-aided detection (CAD) for nodule detection in chest radiography.

**Methods:**

The 140 CXRs (56 with a solitary CT-proven nodules and 84 negative controls) were divided into four subsets of 35; each were read in a different order by six readers. Lesion presence, location and diagnostic confidence were scored without and with CAD (IQQA-Chest, EDDA Technology) as second reader. Readers received individual feedback after each subset. Sensitivity, specificity and area under the receiver-operating characteristics curve (AUC) were calculated for readings with and without CAD with respect to change over time and impact of CAD.

**Results:**

CAD stand-alone sensitivity was 59 % with 1.9 false-positives per image. Mean AUC slightly increased over time with and without CAD (0.78 vs. 0.84 with and 0.76 vs. 0.82 without CAD) but differences did not reach significance. The sensitivity increased (65 % vs. 70 % and 66 % vs. 70 %) and specificity decreased over time (79 % vs. 74 % and 80 % vs. 77 %) but no significant impact of CAD was found.

**Conclusion:**

Short-term feedback does not increase the ability of readers to differentiate true- from false-positive candidate lesions and to use CAD more effectively.

**Key Points:**

• *Computer-aided detection (CAD) is increasingly used as an adjunct for many radiological techniques.*

• *Short-term feedback does not improve reader performance with CAD in chest radiography.*

• *Differentiation between true- and false-positive CAD for low conspicious possible lesions proves difficult.*

• *CAD can potentially increase reader performance for nodule detection in chest radiography.*

## Introduction

Various computer-aided detection (CAD) systems for chest radiography with and without FDA approval have been developed. The latest reported stand-alone sensitivities of these systems for the detection of small focal opacities vary from 34 % to 78 %, depending on the lesion selection and study group [1–6].

Results of studies evaluating the effects of CAD on actual observers' performances are not homogeneous and range from significant improvement [2, 7, 8] to lack of any impact [9]. Results seem to be influenced by the type of CAD algorithm used, reader experience and the conspicuity of the study lesions.

The potential of CAD to increase the radiologist's sensitivity for pulmonary nodules was described earlier: two studies reported that 35 % and 47 % of bronchogenic tumours missed in the original reports were correctly marked by CAD [1, 4]. A third study found a lower agreement between CAD and observers' detection compared with the agreement between observers indicating the ability of CAD to mark lesions the radiologists tend to miss [3]. All three studies, however, compared the CAD performance with previously made observer readings and did not assess the actual influence of CAD on the readers' decisions.

The importance of this interaction between CAD and the observers for a successful implementation of CAD was shown by de Hoop et al. [9]. They had not been able to show a positive effect of the CAD algorithm they tested because readers had difficulties in differentiating true-positive from false-positive CAD candidates. More than two-thirds of the true-positive CAD candidates in whom CAD was given for lesions that were originally missed were not accepted by the readers.

The authors suspected that this inability of the readers to use the CAD more beneficially was at least partly due to a lack of experience with the performance of CAD and subsequently a lack of confidence in the CAD analysis. For CT colonography, it was reported that a 1-day training period already resulted in increased reader sensitivity, but at the expense of decreased specificity and increased reading time [10]. In mammography sensitivity, specificity and area under the receiver-operating characteristics curve (AUC) significantly increased after a 4-week training period [11]. The ultimate learning curve for CAD in mammography has been estimated to be around 2 years but this has never been evaluated in a structured manner [12]. Up to now there have been no studies evaluating the effect of observer training on the application of CAD in chest radiography.

The purpose of the current study was to test whether short-term feedback to readers on their own performance when using CAD would increase the readers' confidence in the CAD analysis and thus their ability to differentiate true- from false-positive CAD candidates.

## Materials and methods

### Study population

For this retrospective study we selected 140 patients from our institution's data archive. Patients were included if a two-view chest radiograph (CXR) and thoracic CT were obtained within 6 weeks and revealed no or a single nodular opacity. The diameter ranged from 5 mm to 15 mm (measured on axial CT images) and none of the nodules showed calcifications. Of the 140 patients 56 had a solitary CT-proven nodule and 84 served as negative controls. Patients with more than one nodular opacity or a pathological feature other than COPD on the CXR were excluded.

Ethics committee approval was obtained and because of the retrospective nature of the study patient informed consent was waived (registration no. 10171150).

### Pulmonary nodules

Thoracic CT served as a reference standard and revealed a solitary nodular opacity in 56 patients (40 %). Conspicuity of the lesion on the CXR was subjectively graded in consensus by a board-certified chest radiologist (>15 years of experience) and the researcher (fifth year resident) who were not involved in the readings and ranged from high (1) to moderate (2), low (3) and very low (4).

### Image acquisition

All CXRs were obtained using a digital technique with a dedicated chest stand (Thoravision Philips Medical Systems, Hamburg, Germany). Images were processed using non-linear multifrequency processing (Unique, Philips Medical Systems, Hamburg, Germany). Processing parameters were implemented following the recommendations of the manufacturer and represented the same as those used as standard processing in our institution. Both the postero-anterior and the lateral views were available for evaluation.

### CAD

We used a commercially available CAD system (IQQA-Chest; EDDA Technology, Princeton Junction, NJ, USA). This system is designed to detect nodules within the range from 5 mm to 15 mm in diameter on the PA radiograph. Images are automatically analysed in the background after acquisition of the images; thus results are immediately available when the radiographs are read but are only shown on demand. The CAD algorithm marks between zero and five suspicious areas with semitransparent circles (candidates).

### Image evaluation

Six observers of vastly varying experience participated in this study: five radiology residents with 0 to 5 years of training (R1, 0 years; R2 and R3, 2 years; R4, 3 years; R5, 5 years) and one board-certified radiologist (R6) with more than 15 years' experience in reading chest films. Two observers (R1 and R3) had no previous experience at all with using CAD in chest radiography; the other four observers had served as observers in previous studies evaluating CAD, two using the same CAD system as in this study and two using a different CAD system. None of the observers had experience with CAD in clinical routine work.

The 140 pairs of PA and lateral radiographs were divided into four subsets of 35 each. Each subset consisted of 14 cases with a solitary nodular opacity and 21 negative control cases. Care was taken that the distribution of nodule conspicuity and the CAD stand-alone sensitivity were equal for the four subsets.

Each observer individually interpreted the 35 PA and lateral chest radiographs of one subset first without and subsequently with the availability of the CAD markings within a single reading session. Observers were asked to determine the presence or absence of an intrapulmonary opacity using a five-point scale of confidence ranging from 5 (pulmonary nodule definitely present) to 3 (equivocal with respect to the presence of a nodule) and 1 (definitely no nodule present). For a confidence rating >1, observers were asked to indicate the anatomical location of the suspected lesion on a separate data sheet. Readings with and without the availability of the CAD results were separately documented. Readers were allowed to modify their confidence levels after CAD also became available for lesions seen during unassisted reading. The observers were informed that images contained only a single nodular opacity and were instructed to ignore calcified lesions and lesions smaller than 5 mm in diameter. Magnification, window/level adjustment and grey-scale reversal were allowed during both readings with and without CAD results.

All observers read the four subsets of CXRs in a different order.

### Feedback on readers' performance

After completion of each of the four subsets of cases, the observer received individual feedback on his/her performance by the researcher. During this feedback, the observer and researcher discussed, on a case by case basis, the location of the lesions if present, the CAD marks with respect to whether they were true- or false-positive and the observer's individual response.

### Data analysis

The stand-alone performance of CAD was determined by calculating the sensitivity and mean false-positive per image (mFP). A one-way ANOVA with Tukey post hoc test was used to test differences among the four subsets.

Sensitivity, specificity and AUC were calculated per observer and per subset for the readings with and without the availability of CAD results. For calculating the sensitivity, ratings 1–3 were considered negative, and ratings 4 and 5 were considered positive if the lesions were correctly localised.

The literature is controversial with regard to whether application of CAD as a second reader should allow for the discharge of lesions located during primary unassisted reading (as done in our study) or should only be used to add potential lesions (add-on mode) [13]. We therefore also analysed the data for the add-on scenario with preservation of all originally indicated lesions. Comparisons of all three methods were made using Cochran Q tests as well as logistic regressions for repeated measurements (GEE). Pairwise comparisons were carried out using McNemar's test for each reader separately. To assess the impact of the feedback on reader performance we compared the results of all readers for the first two subsets with the results for the last two subsets.

All analyses were performed in SPSS 17. Statistical significance was assumed at *P* < 0.05.

## Results

### Study group

Patient mean age was 61 years with no significant difference among the four subsets (59.7, 58.8, 61.9 and 60.3). All subsets consisted of 14 patients with a solitary nodule and 21 negative control patients. Of all nodules 30 % (17) had a high conspicuity, 23 % (13) a moderate, 30 % (17) a low and 16 % (9) a very low conspicuity (Table 1). None of the patients of the diseased or of the control group showed any relevant pathological feature other than the effects of smoking and the focal study lesion.

**Table 1:** Distribution of false-positive (FP) computer-aided detection (CAD) candidates and nodule conspicuity per subset

Subset	Total CAD FP	Nodule conspicuity			
		1	2	3	4
A	56	5	4	4	4
B	70	3	4	3	3
C	52	4	4	5	4
D	82	2	2	2	3

### CAD stand-alone

Stand-alone CAD detected 32 out of 56 nodules leading to a mean sensitivity and sensitivity per subset of 57 %. Sensitivity was 100 %, 54 %, 44 % and 22 % for nodules of high, moderate, low and very low conspicuity, respectively. CAD generated a total of 260 FP candidates with an mFP of 1.9. The mFP for the different subsets amounted to 1.6, 2.0, 1.5 and 2.3 with a significant difference between subsets 3 and 4 (*P* = 0.024). The positive predictive value was 0.31 (32/103), whereas the negative predictive value was 0.35 (13/37).

### Reader performance for subsets 1 and 2

Sensitivity, specificity and AUC were not significantly affected by the use of CAD (Table 1). Mean AUC increased from 0.76 without CAD to 0.78 with CAD, but the difference did not reach significance.

Use of CAD in the add-on scenario led to an increase in sensitivity (65 % vs. 69 %) but at the expense of loss of specificity (79 % vs. 76 %). If discharge of lesion candidates was allowed with the use of CAD, sensitivity changed from 65 % to 66 % and specificity from 79 % to 80 %. None of the differences listed reached statistical significance.

### Reader performance for subsets 3 and 4

Reading sessions 3 and 4, sensitivity, specificity and AUC were not significantly affected by the use of CAD (Table 2). Readers increased their baseline sensitivity to a mean of 70 % at the expense of slightly lower specificity of 74 % compared with the first two reading sessions. With CAD the AUC slightly increased from 0.82 to 0.84, but the differences did not reach significance.

**Table 2:** Averaged reader performance for subsets 1 + 2 and 3 + 4 without and with CAD. Confidence intervals are shown in parentheses

	Sensitivity		Specificity		AUC	
	1 + 2	3 + 4	1 + 2	3 + 4	1 + 2	3 + 4
No CAD	65 % (58–73 %)	70 % (63–77 %)	79 % (74–84 %)	74 % (69–80 %)	0.76 (0.71–0.81)	0.82 (0.78–0.86)
CAD with possible discharge	66 % (59–73 %)	70 % (63–77 %)	80 % (75–85 %)	77 % (72–82 %)	0.78 (0.73–0.83)	0.84 (0.79–0.88)
CAD add-on	69 % (62–76 %)	71 % (65–78 %)	76 % (71–81 %)	74 % (69–80 %)	0.78 (0.73–0.83)	0.85 (0.82–0.89)

*CAD* computer-aided detection

*AUC* area under the receiver operating characteristics curve

CAD with possible discharge: observers were allowed to discharge lesions made during primary unassisted reading

CAD add-on: observers were only allowed to add potential lesions during the reading with CAD

While sensitivity remained unchanged with CAD (70 and 71 %, respectively), specificity slightly increased with CAD when discharge of lesions was allowed (77 and 74 %, respectively). None of the differences listed reached statistical significance.

### Reader-CAD interaction

Analysing pooled data, all six readers dismissed 17 % of the TP CAD candidates (32/192). There was no difference between the first two and last two subsets (16 vs. 16). Of the dismissed TP candidates 50 % (16/32) referred to nodules of low conspicuity and 28 % (9/32) to lesions of very low conspicuity with the remaining 22 % (7/32) referring to moderately conspicuous nodules. The number of dismissed true-positive CAD amounted to 1/1, 0/0, 6/4, 1/0, 1/0 and 1/1 for subsets 1 and 2 versus subsets 3 and 4, respectively for readers 1 to 6.

**Fig. 1:**
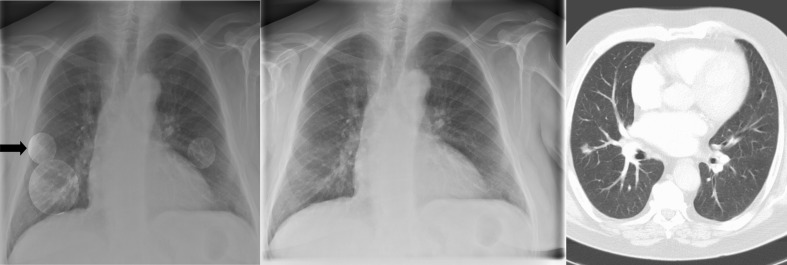
The nodule of low conspicuity in the right lower lobe was missed by three readers without the computer-aided detection (CAD) results. The true-positive CAD candidate was accepted by two readers and dismissed by one reader (*arrow*). None of the readers accepted the false-positive CAD candidates

Based on pooled data analysis the number of accepted FP CAD candidates non-significantly decreased from a total of ten made in the first two readings to six made in the last two readings. An example of reader-CAD interaction is given in Fig. 1.

## Discussion

It is common clinical practice that small primary lung carcinomas are missed on two-view chest radiographs although they were frequently visible in retrospect. Miss rates of between 20 % and 90 % have been reported for primary lung carcinomas [14–20]. Two recent papers reported a CAD stand-alone sensitivity of 35 % and 47 % for pulmonary tumours initially overlooked by the radiologist [1, 4], indicating the potential of CAD to improve the readers' detection performance. Both papers, however, did not include observer performances; thus, to which extent radiologists would have taken advantage of CAD and accepted these true-positive candidates remains unanswered. Equally the risk of accepting false-positive candidates with unnecessary follow-up diagnostics cannot be quantified. The importance of the interaction between the CAD and observer has been previously illustrated by a study that tested the impact of CAD on observer performance for the detection of T1 tumours on chest radiographs of patients who had been part of a CT screening trial: the number of malignancies initially not seen by the observers but correctly annotated by CAD varied between 5 and 16 per observer. However, 80 % of these correctly annotated lesions (46/59) were not accepted by the readers and subsequently dismissed [9]. One underlying reason for the readers' inability to differentiate true- from false-positive lesions might have been the lack of experience with the CAD algorithm and subsequently insufficient trust in its performance.

In the current study we therefore tested the effect of short-term feedback on the detection of pulmonary nodules on digital chest radiography. Our hypothesis was that individual feedback after the interpretation of each of the four subsets would help readers to build up more confidence in the performance of the CAD algorithm, eventually resulting in an increased ability to distinguish true- from false-positive candidates and in a higher acceptance of true-positive CAD candidates.

We found a slight but not significant increase in baseline performance between sessions 1 and 2 compared with sessions 3 and 4, meaning that there was a small overall training effect and that readers detected more nodules in the last two readings than in the first two readings. For none of the paired sessions, however, could we prove a significant influence of reader performance by CAD. Neither the acceptance of true-positive CAD candidates nor the ability to dismiss false-positive candidates was significantly influenced by the feedback information. There was an overall tendency towards increased performance with CAD but the differences were too small to reach significance.

Seventy-eight percent of the dismissed true-positive CAD candidates referred to lesions of low and very low conspicuity, indicating that readers had difficulties in assigning sufficient credibility to CAD candidates, indicating nodules with low conspicuity. Feedback did not help in this respect because the number of dismissed true-positive candidates was the same in sessions 1 and 2 compared with sessions 3 and 4.

There are very few studies in the literature evaluating the impact of training on the use of CAD and those referred for CT colonography and mammography. Results were quite different in the respect that a short, 1-day period of training already affected observer performance in CT colonography as opposed to mammography, which had a lower learning curve requiring at least 4 weeks [10, 11]. It seems that the effect of CAD follows different perception and learning rules in CT vs. radiography, and that the beneficial use of CAD to reduce perception errors of well-defined and more conspicuous lesions (e.g. colon polyps) can be learned faster than the use of CAD to detect lesions of low conspicuity that require differentiation from obscuring background noise (e.g. lesions on mammography). This is also supported by our result that most of the dismissed true-positive CAD candidates referred to lesions of low and very low conspicuity.

It is very likely that lesions of high or moderate conspicuity that have been missed by the readers owing to “inattentional blindness” meaning that the lesions missed during routine reporting take advantage of the availability of CAD more effectively and more easily . Unfortunately it is much more difficult to prove this effect under study conditions because readers tend to analyse the radiographs with an especially high degree of alertness compared to normal conditions. Even though the readers in our study generally had a low level of experience in reading chest radiographs—5 out of 6 were residents—the baseline sensitivity was relatively high with a mean of 65 % for the first two subsets and 70 % for the last two subsets. It is possible that this high baseline sensitivity impeded a further increase in sensitivity with the availability of CAD results.

Lesions of low or very low conspicuity, however, have different diagnostic requirements: correct diagnosis requires not only visual localisation but also correct differentiation from surrounding “anatomical” noise. For this type of lesion, our results suggest that CAD has no significant impact on reader behaviour and short-term feedback has no effect. Whether a longer learning period would be more efficient as postulated for mammography [12] remains to be proven for chest radiography.

Our results also underline the need to further decrease the number of false-positive CAD candidates. A lower number of false-positive candidates will not only decrease reading time but also increase readers' confidence in the reliability of CAD, and will help them to focus on the presence of underlying lesions in the circled areas of interest. The number of false-positive calls provoked by CAD was quite low in this study: 10 and 6, respectively, pooled over all readers for the first two and last two readings.

It might also be the case that the pure presentation of CAD candidates alone is not sufficient and more information is needed to help the reader to correctly differentiate true- from false-positive lesions. In this context the availability of likelihood calculations together with an active localisation procedure by the reader him/herself has been found to be very effective for mammography [21].

Our study suffers from the following limitations. Nodule incidence in the study population was higher than usually seen under clinical conditions. Although the readers did not know the exact distribution of positive and negative cases, they certainly were more alert to detecting focal lesions than in a usual clinical setting.

The number of CAD false-positive candidates was not equally distributed over the four subsets, although we consider it unlikely that this had an effect on our results as all readers interpreted the subsets in a different order and there was a generally low number of accepted CAD false-positive candidates.

This study used a specific type of CAD algorithm. It has to be noted that these results are not necessarily transferrable to other CAD algorithms: a different or updated CAD algorithm with a different performance may yield different results in a context in which perception, reader experience and confidence as well as lesion conspicuity form a complicated framework.

Our study represents the experience of one institution; whether a different reader group or involving readers from various institutions would have yielded different results remains speculative.

We conclude that short-term feedback does not significantly increase the ability of readers to differentiate true- from false-positive candidate lesions in chest radiography in order to use CAD more effectively for the detection of nodular lesions. Further research is needed to determine whether a longer training period or additional processing and display tools such as temporal subtraction, rib suppression or likelihood calculations can increase the benefits of CAD for reader performance in chest radiography.
